# Increasing Risks of Suicide Attempt and Suicidal Drug Overdose After Head Trauma in Patients With Sleep-Disordered Breathing: A Population-Based Study

**DOI:** 10.3389/fpsyt.2020.533784

**Published:** 2020-12-09

**Authors:** Dorji Harnod, Cheng-Li Lin, Tomor Harnod, Chia-Hung Kao

**Affiliations:** ^1^Department of Emergency and Critical Care Medicine, Fu Jen Catholic University Hospital, Fu Jen Catholic University, New Taipei City, Taiwan; ^2^School of Medicine, College of Medicine, Fu Jen Catholic University, New Taipei City, Taiwan; ^3^Management Office for Health Data, China Medical University Hospital, Taichung, Taiwan; ^4^College of Medicine, China Medical University, Taichung, Taiwan; ^5^Department of Neurosurgery, Hualien Tzu Chi Hospital, Buddhist Tzu Chi Medical Foundation, Hualien County, Taiwan; ^6^College of Medicine, Tzu Chi University, Hualien County, Taiwan; ^7^Center of Augmented Intelligence in Healthcare, China Medical University Hospital, Taichung, Taiwan; ^8^School of Medicine, College of Medicine, Graduate Institute of Biomedical Sciences, China Medical University, Taichung, Taiwan; ^9^Department of Nuclear Medicine and Positron Emission Tomography Center, China Medical University Hospital, Taichung, Taiwan; ^10^Department of Bioinformatics and Medical Engineering, Asia University, Taichung, Taiwan

**Keywords:** cohort study, head trauma, national health insurance, sleep-disordered breathing, suicide

## Abstract

**Objective:** To determine the risks of suicide attempt (SA) and suicidal drug overdose (SDO) after head trauma in patients with sleep-disordered breathing (SDB) by using the National Health Insurance Research Database of Taiwan.

**Methods:** We analyzed the data of patients aged ≥20 years who were diagnosed with SDB between 2000 and 2012. We further divided them into two cohorts [with admission for head injury (SBI) and without (SBN)], and we compared them against sex-, age-, comorbidity-, and index-date-matched healthy individuals. The adjusted hazard ratios (aHRs) and 95% confidence intervals of SA and SDO were calculated with adjustment of age, sex, and comorbidities.

**Results:** Approximately 0.61% of patients among the overall 142,063 patients with SDB had SA, with 535 and 335 patients included in the SBN and SBI cohorts, respectively. Compared with patients with SBN, a significantly higher risk of SA was observed in patients with SBI (aHR = 2.22), especially in those aged under 50 years (aHR = 2.48). Notably, a SDO incidence of 1.20% was noted in patients with SDB, and the SBI cohort had a 1.81-fold higher risk for SDO when compared with the SBN cohort.

**Conclusion:** The risks of subsequent SA and SDO are proportionally increased by the effects of head trauma with a moderating role of SDB, especially in those aged <50 years. SDB and head trauma can increase suicide behaviors individually and synergistically.

## Introduction

More than 800,000 people die because of suicide per year worldwide, with a progressive increase over the past few decades (1). In most countries, the rates of suicide attempt (SA) increase with age. Generally, men have a lower rate of SA than women; however, men exhibit higher lethality of suicide than women (1). A study conducted in European countries has estimated the rates of suicidal lethality in men compared with women (13.9 vs. 4.1%) (2). The primary reason for this sex difference is the difference in suicidal methods adopted by men and women. Among various suicidal methods, self-intoxication is the most frequently chosen method in women (3). We believe that identifying individuals with a possibly higher risk of SA and interrupting their suicidal behaviors are the significant challenges of suicide prevention.

Sleep disorders manifesting as dyssomnia or insomnia are common health problems, which affect not only the quality but also the longevity of a patient's life (4, 5). Sleep disorders can be associated with various medical and psychological disorders (5). Among the different types of sleep disorders, sleep apnea or sleep-disordered breathing (SDB) is a significant cause of excessive daytime sleepiness and driving accidents, leading to accidental injuries in patients' daily lives (6). In addition to that, SDB can cause other disorders or traumatic brain insult (TBI) (6, 7); ~2.9% of patients with SDB were reported to have suicidal ideation or suicide planning (8).

Accidental head trauma with TBI would typically result in paralysis, impaired consciousness, cognitive problems, and psychotraumatic stress disorder in addition to causing death. Once patients are discharged after admission for head trauma, they might experience long-term or even lifelong physical and psychological consequences (9–11). These physical and psychological disabilities could interact with the mental disturbances caused by SDB, thereby increasing suicidality in these patients. However, the magnitude of increase in the incidence and risk of SA is ambiguous after head trauma in patients with SDB. Therefore, in this study, we used the National Health Insurance Research Database (NHIRD) in Taiwan to determine the incidence and risk of SA after head trauma in patients with SDB.

## Methods

### Data Source

The NHIRD has included all the medical records of insurants covered by the single-payer National Health Insurance (NHI) program of Taiwan since 1995. The purpose of creating NHIRD was to use research results as a reference for medical and health policies and enhance medical research resources. NHIRD contains all medical information, including ambulatory and inpatient services, of more than 99% of the population in Taiwan (12). We used the hospitalization files from the NHIRD to conduct this study with high validity. Diagnoses and outcomes were coded by physician specialists as per the International Classification of Diseases, Ninth Revision, Clinical Modification (ICD-9-CM). The identification number of every study subject was re-coded before the database was released by the government, and all the information of patients was encrypted for personal privacy. The Research Ethics Committee of China Medical University and Hospital in Taiwan approved this study (CMUH104-REC2-115-CR4).

Taiwan is located in East Asia and has identical mores to that of China and most of the Southeast Asian societies (13). Therefore, our study results could be beneficial for the medical care system in both Taiwan and other Asian countries.

### Study Population

**Figure 1:**
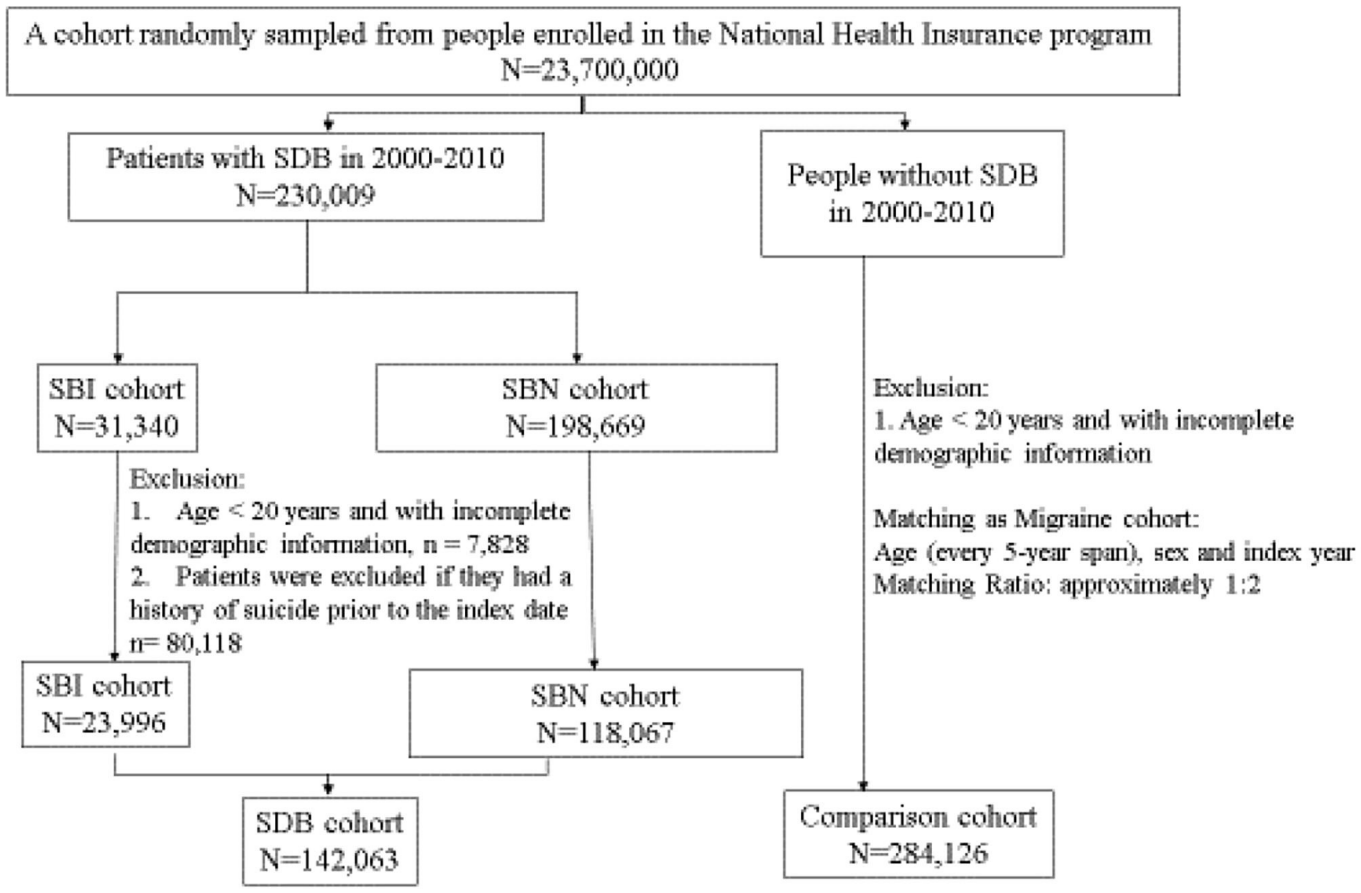
The flowchart of the study population selected from the national health insurance research database.

We enrolled patients who were ≥20 years old to clarify the association between SDB, head trauma, and the risk of SA. We defined the following four cohorts in this study: patients with SDB (ICD-9-CM code 780.5; SDB cohort), patients with SDB who had head trauma with subsequent hospital admission (ICD-9 CM codes 850–854 and 959.01; SBI cohort), patients with SDB without head trauma (SBN cohort) during 2000–2010, as well as a comparison cohort of individuals without SDB and head trauma. Patients with head trauma were defined as those who had moderate or severe head trauma with TBI, because the NHI program does not provide inpatient services to patients who have head trauma but clear consciousness and no intracranial hemorrhage or brain contusion in brain imaging studies. The date of head trauma diagnosis or SDB diagnosis was defined as index dates in the SBI or SBN cohorts. The comparison cohort was frequency matched by sex, age, and index year (Figure 1).

### Outcome Measurement

In this study, the primary outcome was the occurrence of SA (ICD-9-CM codes E950-E959). Suicidal drug overdose (SDO, also called self-poisoning) and SA were individually analyzed in these cohorts to further interpret the risk of SA and the safety of long-term prescription in patients with SDB. Patients with SDO were enrolled for analysis if they were ever hospitalized with the ICD-9-CM codes 960–979 but without E codes. We excluded patients who were younger than 20 years and those who ever had SA or SDO before the index date. Because of their association with SA and SDO, the following comorbidities were evaluated as potential confounders in each cohort: schizophrenia (ICD-9-CM code 295), alcohol-related illness (ICD-9-CM codes 303.0, 303.9, 305.0, 790.3, 980, and E860), anxiety (ICD-9-CM codes 300, 309.24, and 293.84), mental disorder (ICD-9-CM codes 293, 294.0, 294.8, 294.9, and 297), diabetes mellitus (ICD-9-CM code 250), hypertension (ICD-9-CM code 416), hyperlipidemia (ICD-9-CM code 272.4), chronic obstructive pulmonary disease (COPD, ICD-9-CM codes 491, 492, 494, and 496), coronary artery disease (CAD, ICD-9-CM codes 410-413, 414.01–414.05, 414.8, and 414.9), stroke (ICD-9-CM codes 430-438), and cirrhosis (ICD-9-CM codes 571.2, 571.5, and 571.6). These comorbidities were considered confounders for multivariate adjustment in the analysis. All study subjects started follow-up from the index date to the date of SA or SDO occurrence, death, withdrawal from the NHIRD, or December 31, 2013, whichever occurred first.

### Statistical Analysis

Distribution comparisons of categorical variables, such as sex, age, monthly income, urbanization level, occupation, and comorbidities, were performed using univariate analysis with chi-square testing. For continuous variables such as average age, Student's *t*-test was performed to compare the difference between the SDB and comparison cohorts. The urbanization level was divided into four levels based on the population density of the residential area, wherein level 1 was the most urbanized and level 4 was the least urbanized. Occupation was categorized into office worker, laborer, and others (those were defined as primarily retired, unemployed, or from low-income populations). Cox proportional hazard regression models adjusted for age, sex, monthly income, urbanization level, occupation, and comorbidities were applied to assess the risks of SA and SDO in the SDB, SBN, and SBI cohorts relative to the comparison cohort. This study also used Cox regression to compare the risks of SA and SDO between the SBN and SBI cohorts. Hazard ratio (HR), adjusted hazard ratio (aHR), and 95% confidence interval (CI) were calculated to evaluate the risks of SA and SDO. Statistical analyses were conducted with type I error α = 0.05 using the statistical software package, SAS, version 9.4 (SAS Institute, Inc, Cary, NC).

## Results

The baseline demographic characteristics for these cohorts are shown in Table 1. SDB was diagnosed in 142,063 patients between January 1, 2000, and December 31, 2010. Of these, 23,996 (0.17%) patients had SDB and subsequent admission for head trauma, and the remaining 118,067 patients had SDB but without head trauma. The comparison cohort included 284,126 subjects without SDB and head trauma. Among the cohorts, parameters such as male sex, age <50 years, monthly income of NTD 15,000–19,999, living in a level 2 urbanization area, and occupation of office worker were dominant. Notably, patients with SDB demonstrated higher risks of various comorbidities, including schizophrenia, alcohol-related illness, anxiety, mental disorder, diabetes mellitus, hypertension, hyperlipidemia, COPD, CAD, stroke, and cirrhosis (Table 1).

**Table 1:** Distribution of sex, age, monthly income, urbanization level, occupation, and comorbidities between the SDB, SBN, SBI, and comparison cohorts.

	**SDB** ***N*** **=** **142,063**		**SBN** ***N*** **=** **118,067**		**SBI** ***N*** **=** **23,996**		**Comparison** ***N*** **=** **284,126**		
	***n***	**%**	***n***	**%**	***n***	**%**	***n***	**%**	***p*-value^a^**
Sex									0.99
Female	64,589	45.5	54,975	46.6	9,614	40.1	129,178	45.5	
Male	77,474	54.5	63,092	53.4	14,382	59.9	154,948	54.5	
Age (years)									0.99
≤ 49	53,912	38.0	44,320	37.5	9,592	40.0	107,824	38.0	
50–64	36,348	25.6	30,919	26.2	5,429	22.6	72,696	25.6	
≥65	51,803	36.5	42,828	36.3	8,975	37.4	103,606	36.5	
Age, mean ± SD^§^	46.7 ± 19.3		46.7 ± 19.3		46.0 ± 18.1		46.5 ± 19.3		
Monthly income^†^									<0.001
<15,000	41,472	29.2	33,883	28.7	7,589	31.6	77,352	27.2	
15,000–19,999	69,224	48.7	56,416	47.8	12,808	53.4	129,450	45.6	
≥20,000	31,367	22.1	27,768	23.5	3,599	15.0	77,324	27.2	
Urbanization level^‡^									<0.001
1 (highest)	32,994	23.2	29,195	24.7	3,799	15.8	82,186	28.9	
2	42,879	30.2	35,824	30.3	7,055	29.4	84,297	29.7	
3	23,466	16.5	19,382	16.4	4,084	17.0	47,795	16.8	
4 (lowest)	42,724	30.1	33,666	28.5	9,058	37.8	69,848	24.6	
Occupation category^&^									<0.001
Office worker	61,192	43.1	52,903	44.8	8,289	34.5	143,935	50.7	
Laborer	59,901	42.2	48,438	41.0	11,463	47.8	106,993	37.7	
Other	20,970	14.8	16,726	14.2	4,244	17.7	33,198	11.7	
**Comorbidity**									
Schizophrenia	3079	2.17	2,656	2.25	423	1.76	1,232	0.43	<0.001
Alcohol-related illness	8,161	5.74	4,777	4.05	3,384	14.1	1,168	0.41	<0.001
Anxiety	9,909	6.98	7,880	6.67	2,029	8.46	965	0.34	<0.001
Mental disorders	37,717	26.6	28,382	24.0	9,335	38.9	6,909	2.43	<0.001
Diabetes mellitus	25,061	17.6	19,757	16.7	5,304	22.1	18,276	6.43	<0.001
Hypertension	54,100	38.1	43,960	37.2	10,140	42.3	34,659	12.2	<0.001
Hyperlipidemia	18,189	12.8	14,199	12.0	3,990	16.6	8,387	2.95	<0.001
Chronic obstructive pulmonary disease	23,784	16.7	18,332	15.5	5,452	22.7	10,795	3.80	<0.001
Coronary artery disease	26,217	18.5	20,724	17.6	5,493	22.9	16,090	5.66	<0.001
Stroke	25,440	17.9	19,557	16.6	5,883	24.5	14,181	4.99	<0.001
Cirrhosis	19,962	14.1	14,005	11.9	5,957	24.8	8,137	2.86	<0.001

*Chi-square test*;

§ *t-test*;

a *Total SDB cohort vs. comparison cohort*;

† *New Taiwan Dollar (NTD), 1 NTD is equal to 0.03 USD*;

‡ *The urbanization level was divided based on the population density of the residential area into four levels, wherein level 1 was the most urbanized and level 4 was the least urbanized*;

& *Other occupation categories included those who were primarily retired, unemployed, and from low-income populations*.

Table 2 reveals that SA was observed in 215 (0.08%) subjects of the 284,126 individuals in the comparison cohort. Moreover, 870 (0.61%) patients among the 142,063 patients in the SDB cohort had a diagnosis of SA during the 14-year follow-up period, and 535 and 335 patients in the SBN and SBI cohorts developed SA, respectively. Patients <50 years of age in the SDB, SBN, and SBI cohorts had a specifically higher risk of SA when compared with the comparison cohort (aHR = 8.18, 95% CI = 6.09–11.0; aHR = 6.66, 95% CI = 4.93–9.00; aHR = 16.6, 95% CI = 12.0–22.9, respectively). Regarding sex stratification, the SBI cohort specifically had a higher risk of SA compared with the comparison cohort (aHR = 7.18, 95% CI = 5.45–9.46 for men; aHR = 9.96, 95% CI = 7.44–13.3 for women). Patients with SBI and monthly income below NTD15,000 and with NTD15,000–19,999 also specifically had a higher risk of SA (aHR = 8.76, 95% CI = 6.14–12.5, aHR = 8.81, 95% CI = 6.70–11.6, respectively), compared with comparisons. Compared with the comparison cohort, the SDB cohort generally had a higher risk of SA across different urbanization levels, occupations, and with or without comorbidities. However, patients with SBI specifically had a higher risk of SA irrespective of the urbanization level (aHR = 10.3, 9.76, 8.82, and 6.61 for levels 1 to 4) or occupation categories (aHR = 10.5, 7.42, and 6.84 for office worker, laborer, and others, respectively) (Table 2).

**Table 2:** Comparison of incidence and hazard ratio for suicide attempt stratified by age, sex, monthly income, urbanization level, occupation, and comorbidity between the SDB, SBN, SBI, and comparison cohorts.

	**Comparison** ***N*** **=** **284,126**		**SDB** ***N*** **=** **142,063**			**SBN** ***N*** **=** **118,067**			**SBI** ***N*** **=** **23,996**		
	**Event (*N*)**	**Rate^#^**	**Event (*N*)**	**Rate**	**Adjusted HR (95% CI)^$^**	**Event (*N*)**	**Rate**	**Adjusted HR (95% CI)^&^**	**Event (*N*)**	**Rate**	**Adjusted HR (95% CI)^&^**
All	215	1.65	870	14.8	4.75 (4.02, 5.62)^***^	535	11.1	4.09 (3.44, 4.86)^***^	335	31.4	8.44 (6.91, 10.3)^***^
**Age, years**											
≤ 49	58	1.11	497	19.9	8.18 (6.09, 11.0)^***^	264	13.2	6.66 (4.93, 9.00)^***^	233	46.8	16.6 (12.0, 22.9)^***^
50–64	43	1.27	174	11.3	4.68 (3.21, 6.82)^***^	120	9.34	4.27 (2.91, 6.27)^***^	54	21.4	7.38 (4.64, 11.7)^***^
≥65	114	2.58	199	10.7	2.84 (2.17, 3.71)^***^	151	9.78	2.69 (2.04, 3.55)^***^	48	15.1	3.68 (2.52, 5.37)^***^
*P* for interaction					<0.001			<0.001			
**Sex**											
Female	94	1.59	447	16.3	5.52 (4.31, 7.08)^***^	294	12.8	4.76 (3.69, 6.14)^***^	153	33.7	9.96 (7.44, 13.3)^***^
Male	121	1.70	423	13.4	4.11 (3.27, 5.17)^***^	241	9.48	3.53 (2.79, 4.48)^***^	182	29.6	7.18 (5.45, 9.46)^***^
*P* for interaction					0.08			0.35			
**Monthly income^†^**											
<15,000	68	1.96	268	16.1	4.63 (3.43, 6.26)^***^	160	11.9	3.92 (2.87, 5.35)^***^	108	33.7	8.76 (6.14, 12.5)^***^
15,000–19,999	106	1.79	496	17.1	5.30 (4.20, 6.69)^***^	308	13.3	4.62 (3.64, 5.87)^***^	188	32.2	8.81 (6.70, 11.6)^***^
≥20,000	41	1.13	106	7.90	3.13 (2.05, 4.79)^***^	67	5.70	2.75 (1.77, 4.26)^***^	39	23.6	6.42 (3.72, 11.1)^***^
*P* for interaction					0.94			0.18			
**Urbanization level^‡^**											
1 (highest)	34	0.90	141	10.4	5.52 (3.63, 8.38)^***^	98	8.23	4.97 (3.24, 7.60)^***^	43	25.6	10.3 (6.09, 17.3)^***^
2	62	1.60	272	15.3	5.65 (4.15, 7.69)^***^	174	12.0	4.96 (3.63, 6.82)^***^	98	30.8	9.76 (6.75, 14.1)^***^
3	33	1.51	140	14.1	4.52 (2.95, 6.92)^***^	81	9.98	3.76 (2.42, 5.85)^***^	59	32.5	8.82 (5.36, 14.5)^***^
4 (lowest)	86	2.68	317	17.9	3.79 (2.89, 4.96)^***^	182	13.3	3.16 (2.38, 4.18)^***^	135	33.6	6.61 (4.82, 9.06)^***^
*P* for interaction					0.008			<0.001			
**Occupation category^&^**											
Office worker	77	1.16	319	12.4	5.77 (4.38, 7.61)^***^	211	9.58	5.12 (3.86, 6.79)^***^	108	28.7	10.5 (7.49, 14.8)^***^
Laborer	99	2.01	402	16.2	4.37 (3.41, 5.60)^***^	242	12.3	3.72 (2.88, 4.81)^***^	160	31.1	7.42 (5.55, 9.93)^***^
Other	39	2.64	149	17.8	3.57 (2.38, 5.35)^***^	82	28.7	2.92 (1.91, 4.44)^***^	67	37.8	6.84 (4.27, 11.0)^***^
*P* for interaction					0.02			0.006			
**Comorbidity**^§^											
None	117	1.08	149	7.16	6.59 (5.13, 8.46)^***^	105	5.79	5.41 (4.13, 7.09)^***^	44	16.4	14.0 (9.76, 20.0)^***^
With any one	98	4.46	721	18.9	2.97 (2.39, 3.69)^***^	430	14.3	2.37 (1.89, 2.96)^***^	291	36.3	5.32 (4.19, 6.76)^***^
*P* for interaction					0.009			<0.001			

*CI, confidence interval; HR, hazard ratio; PY, person-years*;

# *Incidence rate per 10,000 person-years*;

$ *Multivariable analysis included age, monthly income, urbanization level, and comorbidity of schizophrenia, alcohol-related illness, anxiety, mental disorders, diabetes mellitus, hypertension, hyperlipidemia, chronic obstructive pulmonary disease, coronary artery disease, stroke, and cirrhosis*;

† *New Taiwan Dollar (NTD), 1 NTD is equal to 0.03 USD*;

‡ *The urbanization level was divided based on the population density of the residential area into four levels, wherein level 1 was the most urbanized and level 4 was the least urbanized*;

& *Other occupation categories included those who were primarily retired, unemployed, and from low-income populations*;

§ *Individuals with schizophrenia, depression, alcohol-related illness, anxiety, mental disorders, diabetes mellitus, hypertension, hyperlipidemia, chronic obstructive pulmonary disease, coronary artery disease, stroke, and cirrhosis were classified into the comorbidity group*;

*** *p < 0.001*.

Table 3 presents the incidence and HR of SA between the SBN and SBI cohorts. Compared with patients with SBN, a significantly higher risk of SA was observed in patients with SBI (aHR = 2.22), especially in those aged under 50 years (aHR = 2.48). Generally, patients with SBI who were in all difference stratified had a significantly higher risk of SA when compared with patients with SBN (Table 3).

**Table 3:** Comparison of incidence and hazard ratio for suicide attempt stratified by age, sex, and comorbidities between patients with SDB with and without head trauma.

	**SBN (*N* = 118,067)**	**SBI (*N* = 23,996)**
	**Adjusted HR^$^ (95% CI)**	**Adjusted HR^&^ (95% CI)**
All	1.00	2.22 (1.92, 2.56)^***^
**Age, years**		
≤ 49	1.00	2.48 (2.06, 3.00)^***^
50–64	1.00	1.83 (1.31, 2.56)^***^
≥65	1.00	1.47 (1.05, 2.04)^*^
*P* for interaction		<0.001
**Sex**		
Female	1.00	2.10 (1.72, 2.58)^***^
Male	1.00	2.06 (1.68, 2.54)^***^
*P* for interaction		0.26
**Monthly income^†^**		
<15,000	1.00	2.29 (1.76, 2.96)^***^
15,000–19,999	1.00	1.90 (1.57, 2.30)^***^
≥20,000	1.00	2.47 (1.61, 3.79)^***^
*P* for interaction		0.20
**Urbanization level^‡^**		
1 (highest)	1.00	2.10 (1.44, 3.07)^***^
2	1.00	1.98 (1.52, 2.57)^***^
3	1.00	2.35 (1.64, 3.35)^***^
4 (lowest)	1.00	2.06 (1.63, 2.60)^***^
*P* for interaction		0.42
**Occupation category^&^**		
Office worker	1.00	2.09 (1.63, 2.67)^***^
Laborer	1.00	1.97 (1.60, 2.43)^***^
Other	1.00	2.39 (1.70, 3.37)^***^
*P* for interaction		0.79
**Comorbidity^§^**		
None	1.00	2.63 (1.84, 3.77)^***^
With any one	1.00	2.25 (1.94, 2.63)^***^
*P* for interaction		0.50

*CI, confidence interval; HR, hazard ratio*;

$ *Multivariable analysis included age, monthly income, urbanization level, and comorbidities of schizophrenia, alcohol-related illness, anxiety, mental disorders, insomnia, diabetes mellitus, hypertension, hyperlipidemia, chronic obstructive pulmonary disease, coronary artery disease, stroke, and cirrhosis*;

† *New Taiwan Dollar (NTD), 1 NTD is equal to 0.03 USD*;

‡ *The urbanization level was divided based on the population density of the residential area into four levels, wherein level 1 was the most urbanized and level 4 was the least urbanized*;

& *Other occupation categories included those who were primarily retired, unemployed, and from low-income populations*;

§ *Individuals with schizophrenia, depression, alcohol-related illness, anxiety, mental disorders, and insomnia were classified into the comorbidity group*;

* *p < 0.05*,

*** *p < 0.001*.

Table 4 demonstrates the overall incidence of SDO in the comparison cohort and the SDB, SBN, and SBI cohorts. Of 142,063 patients with SDB, 1,121 patients with SBN and 585 patients with SBI were diagnosed with SDO, with a total incidence of 1.20%. By contrast, 596 patients had SDO among the 284,126 individuals in the comparison cohort, with an incidence of 0.21%. Compared with the comparison cohort, the SDB cohort (aHR = 3.38, 95% CI = 3.04–3.76), SBN cohort (aHR = 2.99, 95% CI = 2.68–3.34), and SBI cohort (aHR = 5.36, 95% CI = 4.69–6.12) had a significantly higher risk of SDO after adjusting for confounders. Upon comparing the SBI and SBN cohorts, the SBI cohort had a significantly higher risk of SDO, with aHR of 1.81 (95% CI = 1.63–2.02) (Table 4).

**Table 4:** Incidence of suicidal drug overdose (per 10,000 person-years) and hazard ratios estimated through the Cox method in patients with SDB with or without head trauma.

	**Comparison**	**SDB**	**SBN**	**SBI**
**Variable**	***N* = 284,126**	***N* = 142,063**	***N* = 118,067**	***N* = 23,996**
**Person-years**				
Event, *n*	596	1,706	1,121	585
Rate^#^	4.57	29.0	23.3	55.2
Crude HR (95% CI)	1 (Reference)	6.27 (5.72, 6.89)^***^	5.00 (4.53, 5.53)^***^	12.2 (10.9, 13.6)^***^
Adjusted HR^$^ (95% CI)	1 (Reference)	3.38 (3.04, 3.76)^***^	2.99 (2.68, 3.34)^***^	5.36 (4.69, 6.12)^***^
Crude HR (95% CI)			1 (Reference)	2.45 (2.22, 2.71)^***^
Adjusted HR^$^ (95% CI)			1 (Reference)	1.81 (1.63, 2.02)^***^

# *Incidence rate per 10,000 person-years*;

$ *Multivariable analysis included age, monthly income, urbanization level, and comorbidities of schizophrenia, alcohol-related illness, anxiety, mental disorders, insomnia, diabetes mellitus, hypertension, hyperlipidemia, chronic obstructive pulmonary disease, coronary artery disease, stroke, and cirrhosis*.

*** *p < 0.001*.

Table 5 presents the incidence and HRs of SDO with drug or medicinal substance, SDO with benzodiazepine-based tranquilizers, or SDO with unknown others for the SDB, SBN, and SBI cohorts. Among the categories of SDO, drug or medicinal substance was the method of choice for 293 patients-−57 among 284,126 individuals of the comparison cohort, 137 among 118,067 patients in the SBN cohort, and 99 among 23,996 patients in the SBI cohort. The SDB, SBN, and SBI cohorts had higher risks of drug or medicinal substance overdose than the comparison cohort (aHR = 4.16, 95% CI = 3.00–5.78; aHR = 3.45, 95% CI = 2.46–4.83; aHR = 8.14, 95% CI = 5.56–11.9, respectively). Of the 520 patients who used benzodiazepine-based tranquilizers, 111 were from the comparison cohort, 288 were from the SBN cohort, and 121 were from the SBI cohort, with aHRs of 4.75 (95% CI = 3.75–6.01), 4.42 (95% CI = 3.48–5.62), and 6.55 (95% CI = 4.87–8.80) in SDB, SBN, and SBI cohorts, respectively. Further, the SBI cohort did exhibit a significantly increased risk of SDO with drug or medicinal substance and benzodiazepine-based tranquilizers when compared with the SBN cohort (Table 5).

**Table 5:** Incidence of different ways of suicidal drug overdose (per 10,000 person-years) and hazard ratios estimated through the Cox method in patients with SDB with or without head trauma.

	**Comparison**	**SDB**	**SBN**	**SBI**
**Variable**	***N* = 284,126**	***N* = 142,063**	***N* = 118,067**	***N* = 23,996**
**Poisoning by unspecified drug or medicinal substance**				
Event, *n*	57	236	137	99
Rate	0.44	4.02	2.84	9.34
Crude HR (95% CI)	1 (Reference)	9.06 (6.79, 12.1)^***^	6.38 (4.68, 8.69)^***^	21.5 (15.6, 29.8)^***^
Adjusted HR^$^ (95% CI)	1 (Reference)	4.16 (3.00, 5.78)^***^	3.45 (2.46, 4.83)^***^	8.14 (5.56, 11.9)^***^
Crude HR (95% CI)			1 (Reference)	3.38 (2.61, 4.38)^***^
Adjusted HR^$^ (95% CI)			1 (Reference)	2.39 (1.82, 3.14)^***^
**Poisoning by benzodiazepine-based tranquilizers**				
Event, *n*	111	409	288	121
Rate	0.85	6.96	5.98	11.4
Crude HR (95% CI)	1 (Reference)	8.07 (6.55, 9.96)^***^	6.90 (5.54, 8.59)^***^	13.5 (10.4, 17.5)^***^
Adjusted HR^$^ (95% CI)	1 (Reference)	4.75 (3.75, 6.01)^***^	4.42 (3.48, 5.62)^***^	6.55 (4.87, 8.80)^***^
Crude HR (95% CI)			1 (Reference)	1.96 (1.58, 2.42)^***^
Adjusted HR^$^ (95% CI)			1 (Reference)	1.48 (1.19, 1.86)^***^
**Poisoning by others**				
Event, *n*	428	1,061	696	365
Rate	3.28	18.1	14.5	34.4
Crude HR (95% CI)	1 (Reference)	5.44 (4.86, 6.08)^***^	4.32 (3.83, 4.88)^***^	10.6 (9.19, 12.2)^***^
Adjusted HR^$^ (95% CI)	1 (Reference)	2.92 (2.57, 3.33)^***^	2.57 (2.25, 2.94)^***^	4.64 (3.94, 5.46)^***^
Crude HR (95% CI)			1 (Reference)	2.48 (2.18, 2.81)^***^
Adjusted HR^$^ (95% CI)			1 (Reference)	1.83 (1.60, 2.09)^***^

$ *Multivariable analysis included age, monthly income, urbanization level, and comorbidities of schizophrenia, alcohol-related illness, anxiety, mental disorders, insomnia, diabetes mellitus, hypertension, hyperlipidemia, chronic obstructive pulmonary disease, coronary artery disease, stroke, and cirrhosis*;

*** *p < 0.001*.

## Discussion

Despite recognizing the importance of reducing suicidal deaths worldwide, several developed countries have not yet invested sufficient resources in research and prevention of SA (3). Notably, there was dissociation between knowing suicidal ideation and attempt in patients with emotional or physical distress to prevent their SA efficiently (14). We observed that in the comparison cohort and the cohort of SDB without head trauma, the respective incidence of SA was 1.11 and 13.2 per 10,000 person-years, 1.27 and 9.43 per 10,000 person-years, and 2.58 and 9.78 per 10,000 person-years for individuals <50 years old, 50–64 years old, and ≥65 years old, respectively. Contrary to the general belief that older people with more somatic disorders would develop a higher risk of SA, patients younger than 50 years with SDB were observed to have a much higher risk of SA in this study. Moreover, no apparent different risks for SA were observed between male and female patients in this study.

SDO or self-intoxication is currently the frequently chosen suicidal method in Europe. Approximately 71% of female registered SAs are because of SDO, whereas 50% of male registered SAs are SDO (3). Fortunately, in Europe, more than 95% of people who attempt SDO would eventually survive (2). Although SDO is a non-violent suicidal method with a low mortality risk than the suicides through violent means, SA with SDO should be effectively prevented before a patient even attempts it, especially in Asian countries (15, 16). This study evidenced the proportional increases in the risks of SA and SDO in the SDB and SBI cohorts. The results implied that prescribed medical substances and tranquilizers could be dangerously abused for suicide (by overdosing on medications) in patients with SDB, even by using the prescription drugs from the medical care system. It should be due to the restricted access to firearms by law in Taiwan and most Asian countries. Therefore, prescription for several medications should hereafter be carefully dispensed in patients with SDB and head trauma.

SDB is known to be typically under-diagnosed and under-treated, and it has strong adverse effects on traffic and work accidents (17). Compared with a study in the United States that reported that 2.81% of adults with abnormal sleep had various degrees of accidental injuries (17), this study revealed a relatively higher incidence (16.89%) of Taiwan residents with SDB who were admitted for moderate or severe head trauma in the 14-year duration. Those with SDB and head trauma were predominantly noted in those <50 years old. The incidence of head trauma and the prevalence of young age in patients with SDB had not been considered significant earlier. Nonetheless, SDB itself could increase the HRs of SA and SDO 4.75 and 3.38 times compared with healthy individuals. After patients with SDB experience head trauma, their HRs of SA and SDO considerably increase to 8.44 and 5.36 times. Therefore, we suggest that head trauma can generally increase the risks of SA and SDO in patients with SDB. Any such admission in Taiwan should be treated with more prudence by clinicians and social workers to prevent patient suicidality, especially in men <50 years old.

Nonetheless, the actual mechanisms of the effects of SDB and admission for head trauma on suicidality and how these mechanisms together increase the risks of SA and SDO warrant further investigational studies. Considering the neuro-psychological pathology of psychotrauma and TBI, there were biological evidences that brain insult could increase the neuro-inflammation reactions involving the blood–brain barrier, glutamate regulation, microglia activation, and autoimmune response (18, 19), which can affect patients' physical endurance, sleep, chronic cognition, and associated psychosocial sequelae through the dysregulation of cortical reactivity (20–22). These pathologies would lead to a pre- and post-TBI gap in re-engaging patients' desired lives after head trauma, thereby contributing to suicidality. However, the relationship between SDB and suicidality is a topic of debate as per current studies. Oxidative stress because of intermittent hypoxemia of SDB (23) and decreased serotonin synthesis owing to the effects of hypoxemia on tryptophan hydroxylase can probably cause suicidality (24). To the best of our knowledge, only one study specifically examined the relationship between SDB, trauma, and suicidality (25). This study revealed a notably bidirectional relationship between SDB severity in posttraumatic patients and their suicidal ideation, but without any information regarding SA in patients.

Our study has documented that the risks of subsequent SA and SDO are proportionally increased by the effects of head trauma with a moderating role of SDB. The study is the first to analyze SA and SDO associated with SDB and head trauma. However, there were several limitations in this study. First of all, this was a retrospective study using the NHI inpatient data related to head trauma and SDB. We identified SA, SDO, head trauma, and SDB based on the inpatient records of the ICD-9-CM coding system. The accuracy of NHI claims data is assured by the severe penalty if fraud and false claiming is found. However, a rare chance of minor SA, SDO, and head trauma in patients with SDB not being enrolled for exists, which can result in little underestimation of the risks of SA and SDO. Second, we could not directly contact patients to understand the details of their disease severity of SDB and head trauma, their suicidal ideation, or treatment for their disorders because patients' identities were anonymized in the NHIRD. These details could be confounding to the individual severity of personal stress and suicidality. Finally, even though the study design controlled numerous confounders as we have already known, some unknown confounders may not be measured. However, this large-scale, unselective study has helped us to understand more about the risks and correlated risk factors for SA and SDO in patients with SDB with and without head trauma, thereby helping to build a more effective prevention system in the future.

## Conclusions

The risks of subsequent SA and SDO are proportionally increased by the effects of head trauma with a moderating role of SDB, especially in those aged <50 years. SDB and head trauma would raise SA and SDO individually and synergistically. Furthermore, our findings provide crucial information that long-term medical substances or tranquilizers in patients with SDB and head trauma should be prescribed with prudence in the future.

## Data Availability Statement

The datasets analyzed in this article are not publicly available. Requests to access the datasets should be directed to the Taiwan Ministry of Health and Welfare (MOHW). The Ministry of Health and Welfare must approve our application to access this data. Any researcher interested in accessing this dataset can submit an application form to the Ministry of Health and Welfare requesting access. Please contact the staff of MOHW (Email: stcarolwu@mohw.gov.tw) for further assistance. All relevant data are within the paper.

## Ethics Statement

The studies involving human participants were reviewed and approved by the Research Ethics Committee of China Medical University and Hospital in Taiwan approved this study (CMUH104-REC2-115-CR4). Written informed consent for participation was not required for this study in accordance with the national legislation and the institutional requirements.

## Author Contributions

TH and C-HK: conception/design. C-HK: provision of study material and patients. All authors: collection and assembly of data, data analysis and interpretation, manuscript writing, and final approval of manuscript.

## Conflict of Interest

The authors declare that the research was conducted in the absence of any commercial or financial relationships that could be construed as a potential conflict of interest.

## Abbreviations

aHR: adjusted hazard ratio
CI: confidence interval
NHIRD: National Health Insurance Research Database
ICD-9-CM: International Classification of Diseases, Ninth Revision, Clinical Modification.
